# Polymorphisms of adiponectin gene and gene–lipid interaction with hypertension risk in Chinese coal miners: A matched case-control study

**DOI:** 10.1371/journal.pone.0268984

**Published:** 2022-09-12

**Authors:** Xiaoqin Hu, Yanfeng Xi, Wenqi Bai, Zhenjun Zhang, Jiahao Qi, Liang Dong, Huiting Liang, Zeyu Sun, Lijian Lei, Guoquan Fan, Chenming Sun, Cheng Huo, Jianjun Huang, Tong Wang

**Affiliations:** 1 Cancer Hospital Affiliated to Shanxi Medical University/Shanxi Hospital Affiliated to Cancer Hospital, Chinese Academy of Medical Sciences/Shanxi Province Cancer Hospital, School of Public Health, Shanxi Medical University, Taiyuan, China; 2 Cancer Hospital Affiliated to Shanxi Medical University/Shanxi Hospital Affiliated to Cancer Hospital, Chinese Academy of Medical Sciences/Shanxi Province Cancer Hospital, Taiyuan, China; 3 School of Public Health, Shanxi Medical University, Taiyuan, China; 4 Sinopharm Tongmei General Hospital, Datong, China; MAHSA University College, MALAYSIA

## Abstract

**Objective:**

Low serum adiponectin level can predict hypertension development, and adiponectin gene (*ADIPOQ*) polymorphisms have been reported to be linked with hypertension risk. Whereas, the interaction between *ADIPOQ* polymorphisms and environmental factors on the susceptibility of hypertension remained unclear. The purpose of this study was to explore the relationship of *ADIPOQ* polymorphisms with hypertension risk and their interaction with lipid levels in coal miners.

**Methods:**

A matched case-control study with 296 case-control pairs was performed in a large coal mining group located in North China. The participants were questioned by trained interviewers, and their *ADIPOQ* genotype and lipid levels were determined. Logistic regression, stratified analysis, and crossover analysis were applied to evaluate the effects of rs2241766, rs1501299, and rs266729 genotypes and gene–lipid interaction on hypertension risk.

**Results:**

In this matched case-control study, the genotypes of rs2241766 TG+GG, rs1501299 GT+TT, and rs266729 CG+GG were marginally related to hypertension risk. Individuals with high total cholesterol (TC), low-density lipoprotein cholesterol (LDL-C), and high-density lipoprotein cholesterol (HDL-C) level were susceptible to hypertension (TC: odds ratio [*OR*] = 1.807, *95%* confidence intervals [*95%CI*] = 1.266–2.581; LDL-C: *OR* = 1.981, *95%CI* = 1.400–2.803; HDL-C: *OR* = 1.559, *95%CI* = 1.093–2.223). Antagonistic interactions were detected between rs2241766 and TC, rs1501299 and TC, rs2241766 and LDL-C, and rs1501299 and HDL-C (rs2241766 and TC: *OR* = 0.393, *95%CI* = 0.191–0.806; rs1501299 and TC: *OR* = 0.445, *95%CI* = 0.216–0.918; rs2241766 and LDL-C: *OR* = 0.440, *95%CI* = 0.221–0.877; rs1501299 and HDL-C: *OR* = 0.479, *95%CI* = 0.237–0.967). Stratified analysis showed that hypertension risk was high for the subjects with rs2241766 TG+GG or rs1501299 GG under the low lipid level but low for those under the high lipid level. In the case group, the TC and LDL-C levels for rs2241766 TG+GG were lower than those for rs2241766 GG, and the TC and HDL-C levels for rs1501299 GT+TT were higher than those for rs1501299 GG.

**Conclusions:**

Although the effects of *ADIPOQ* polymorphisms alone were not remarkable, an antagonistic interaction was observed between *ADIPOQ* polymorphisms and lipid levels.

## Introduction

Hypertension is a crucial worldwide public-health problem because of its high prevalence and concomitant risks of coronary heart disease, stroke, congestive heart failure and renal dysfunction. According to the World Health Organization (WHO), this disease accounts for 7.5 million deaths and 57 million disability adjusted life years (DALYs) [1]. With the rapid economic development and urbanization of China, the prevalence of hypertension has substantially increased. According to the National Chronic Disease and Risk Factor Surveillance of 194 779 adults in China in 2018, hypertension had a prevalence of 27.5% [2] and resulted in serious health implications within the population, including occupational populations in mining areas. Owing to their prolonged underground exposure, coal miners have a high risk of hypertension and other cardiovascular events. Approximately 50% of mining excavator operators were diagnosed with temporary hypertension within a decade [3]. Nevertheless, under the same situation, coal miners were always in better health and had a decreased risk of cardiovascular and cerebrovascular mortality than the general population [4]. The pathogenesis of hypertension in coal miners is extremely complex.

Adiponectin is an adipose tissue-derived cytokine that increases insulin sensitivity by enhancing lipid β-oxidation in skeletal muscles and reducing hepatic gluconeogenesis [5, 6]. Its low circulating level is linked to obesity, diabetes, and hypertension [7–9]. Hypertensive patients exhibit a decrease in plasma adiponectin level, suggesting the role of this hormone in the pathogenesis of hypertension. The adiponectin gene (*ADIPOQ*), which is located on chromosome 3q27, encodes adiponectin. A meta-analysis showed that hypertensive adults have 1.64 μg/mL lower adiponectin levels than normotensive adults, and every 1 μg/mL increase in adiponectin level is associated with a 6% reduction in hypertension risk [10]. The effects of adiponectin on the cardiovascular system are partially mediated by the activation of 5ʹ adenosine monophosphate-activated protein kinase (AMPK) and cyclooxygenase-2 (COX-2) pathways, reduction in endothelial cell apoptosis, promotion of nitric oxide production, and decrease in tumor necrosis factor-alpha (TNF-α) activity [11]. All these findings imply that *ADIPOQ* might be linked to the presence of hypertension. After conducting an extensive article review, we chose three of the most studied hotspot loci on *ADIPOQ* to investigate the association between gene polymorphisms of adiponectin and the susceptibility to hypertension. These single nucleotide polymorphisms (SNPs) are *ADIPOQ* +45 T > G (rs2241766), +276 G > T (rs1501299), and -11377 C > G (rs266729), which are related to adiponectin level [12, 13], commonly found in Chinese individuals (all minor allele frequency (MAF)>0.2, reported from https://pubmed.ncbi.nlm.nih.gov), and therefore could be useful as markers for genetic association studies.

Hypertension is a chronic disease caused by a complex interplay of genetic and environmental risk factors. Thus, to explore the interactions between genes and environmental factors may provide new insights for hypertension etiology. In addition to regulating insulin sensitivity and blood glucose levels, adiponectin controls lipid metabolism. Animal and human studies suggested that adiponectin is implicated in the pathogenic mechanisms of dyslipidemia [14, 15], which is related to hypertension [16, 17]. A number of cohort studies indicated a causal relationship between dyslipidemia and risk of hypertension [18–21]. Thus, this study selected serum lipid levels (including total cholesterol [TC], low-density lipoprotein cholesterol [LDL-C], high-density lipoprotein cholesterol [HDL-C], and triglyceride [TG]) as potential environmental interaction factors.

A matched case-control study was conducted to elucidate the relationship of *ADIPOQ* polymorphism with hypertension risk and its possible interaction with lipid levels in coal miners.

## Materials and methods

### Study population

Participants were recruited from the Datong Coal Mine Group, which is located in the north of Shanxi Province and has approximately 200,000 permanent staff members in 87 coal mines. The administrative department of the coalmine group provided the baseline data that included gender, date of birth, work type, and name of coal miners for the development of the sampling frame. According to the targets, a two-stage cluster sampling was employed to select the participants. In the first phase, 10 coal mines were randomly sampled from 87 coal mines of three coal group areas (Pingwang Region, Kouquan Trench, and Yungang Trench) as the primary sampling unit; these sampled coal mines have 38,951 permanent staff members. In the second stage, stratified random sampling was applied on the basis of several factors including work place (underground or ground), age (20–65 years, with groups of 5-year intervals), and gender (male or female). For the cross-sectional study, the sample size was calculated with a prevalence of hypertension of 30.17% (obtained from 2003 survey in Datong Coal Mine Group), an allowable error of 0.015, and *α* type I error of 0.05. Hence, a sample size of 3650 deliveries was necessary. In considering of the potential of lost to follow up and withdraw from the study, the sample size was expanded by 20% and finally determined to be 4380. The respondents who could not attend the survey due to off duty or other reasons would be notified by the coordination group to make up the survey the next day. At last, a total of 4341 miners were asked to complete questionnaires and provide blood samples from August 1, 2013 to December 30, 2013. For the case-control study, the sample size was calculated with an expected mutation rate of 30%, an estimated odds ratio of 1.5 for SNP on the hypertension risk, *α* type I error of 0.05, and *β* type II error of 0.2. Hence, a sample size of 576 deliveries was necessary. In consideration of insufficient blood samples for some individuals, the sample size was determined to be 600. Finally, 592 blood samples of participants (296 hypertensive patients and 296 control subjects) were subjected to genotyping. Random sampling and matching were conducted by SAS 9.2 (SAS Institute Incorporated, Cary, North Carolina, United States), and sample size was calculated by PASS 15 (NCSS Limited Liability Company, Kaysville, Utah, United States).

The participants were asked to rest quietly for at least 5 minutes and avoid exercise and caffeinated beverages for at least 30 minutes prior to blood pressure measurement. Hypertension was defined referencing to the 2010 Chinese guidelines for the management of hypertension [22]: without antihypertensive drug treatment, the systolic blood pressure (SBP) is ≥140 mmHg and/or the diastolic blood pressure (DBP) is ≥90 mmHg. The blood pressure was measured three times at two minutes intervals, and the mean of the three readings was served as the final result. Patients with a history of hypertension and currently using antihypertensive drugs were diagnosed as hypertensive regardless of their blood pressure level. Participants with no antihypertensive drugs treatment, SBP <140 mmHg, and DBP <90 mmHg served as the non-hypertension group. For the case-control study, cases were randomly sampled from the hypertension group, and controls were random sampled from the non-hypertension group. These individuals were matched 1:1 by gender, age (± 2 years), and work place upon enrollment. Exclusion criteria were having secondary hypertension or insufficient blood samples for deoxyribonucleic acid (DNA) extraction.

Written informed consent for an interview and peripheral whole blood were obtained from each study participant. The study protocol was approved by the ethics committees of Shanxi Medical University (the ethics approval number: HX201201).

### Exposure to environmental factors

Interviewers with medical knowledge used a structured and validated questionnaire to collect information from subjects by face-to-face interview and followed a written protocol to ascertain and reduce monitoring, interviewer, and recall bias. All investigators underwent training for the purpose and significance of the research, the explanation of each item in the questionnaire, and the survey methods and passed an evaluation prior to appointment. The questionnaire focused on demographic features and potential hypertension risk factors including age, gender, marital status, education, work place, work category, work duration (current employment), family history of hypertension (i.e., among first- and second-degree relatives), alcohol-drinking habit, smoking habit, body mass index (BMI), and lipid levels (including TC, LDL-C, HDL-C, and TG levels). Alcohol drinking was defined as drinking alcohol (at least 300 mL of beer or 50 g of liquor per time) more than once a week for at least 6 months, and smoking was described as smoking more than one cigarette a day for at least 6 months, currently or before. Lipid levels were classified in accordance with the 2016 Chinese guideline for the management of dyslipidemia in adults [23]. High TC level was defined as ≥5.18 mmol/L, and <5.18 mmol/L was acceptable. High LDL-C level was defined as ≥3.12 mmol/L, and <3.12 mmol/L was acceptable. Low HDL-C level was defined as <1.04 mmol/L, and ≥1.04 mmol/L was acceptable. High TG level was defined as ≥1.7 mmol/L, and <1.7 mmol/L was acceptable. The missing number for the variables included in the study ranged from 0 to 18. The variables of age, gender, work place, family history of hypertension, TC level, LDL-C level, HDL-C level, and TG level had none missing value, and work duration had the most missing values (3.04%, 18/592). Missing data were filled according to the average value of other participants with the same gender, age, workplace, and other information.

### Blood sample collection and analysis

The cases and controls were requested to provide peripheral whole blood collected in ethylenediaminetetraacetic acid tubes after overnight fasting (>8 h). Genomic DNA was extracted from 200 μL of each blood sample using QIAamp DNA Blood Mini Kit (#51104; Qiagen, Valencia, California, United States) in accordance with the manufacturer’s instructions. Kompetitive Allele Specific Polymerase Chain Reaction (KASP) (Applied by Gene Company Limited, Beijing, China) was used to detect the genotypes of rs2241766, rs1501299, and rs266729. The primer sequences for the KASP of the three SNPs are shown in Table 1. Genotyping reactions were performed in a Hydrocycler-16 (Laboratory of the Government Chemist [LGC] Genomics, United Kingdom) in a final volume of 1 μL containing 0.5 μL of 2×KASP 1536 Master Mix (LGC Genomics, United Kingdom), 0.014 μL of primer mix (prepared as recommended by LGC [46 μL of ddH_2_O, 30 μL of 100 μM common primer and 12 μL of 100 μM each tailed primer and approximately 10 ng of genomic DNA]). The following cycling conditions were used: hot start at 94°C for 10 min, followed by 10 touchdown cycles (94°C for 20 s, touchdown 61°C, −0.6°C per cycle, 10 s) and 26 cycles of amplification (94°C for 20 s, touchdown 61°C, −0.6°C per cycle, 10 s). Since the KASP amplicons are usually smaller than 120 bp, no extension step is necessary in the polymerase chain reaction (PCR) protocol. Fluorescence detection of the reactions was performed using Pherastar scanner (LGC Genomics, United Kingdom), and the data were analyzed using Kraken software (LGC Genomics, United Kingdom). If the signature genotyping groups had not formed after the initial amplification, then additional amplification cycles (usually 5–10) were applied, and the samples were read again. Three percent of the duplicate samples were used to test the accuracy of the genotyping results. DNA extraction and genotyping were conducted between September 2018 and January 2019. Serum TC, LDL-C, HDL-C, and TG levels were determined on the day of blood collection by routine enzymatic methods on automated modular analysersanalyzers (Siemens Advia 2400 Chemistry Analyser, Diamond Diagnostic, Holliston, Massachusetts, United States) at the General Hospital of the Datong Coal Mining Group (Datong, Shanxi, China).

**Table 1:** Primer sequences for the Kompetitive Allele Specific Polymerase Chain Reaction (KASP) of rs2241766, rs1501299, and rs266729.

Gene	Primer sequence
rs2241766	Forward1: 5’-GCTATTAGCTCTGCCCGGG-3’
	Forward1: 5’-ACTGCTATTAGCTCTGCCCGGT-3’
	Reverse: 5’-CTTGAGTCGTGGTTTCCTGGTCAT-3’
rs1501299	Forward1: 5’-GTGTCTAGGCCTTAGTTAATAATGAATGA-3’
	Forward1: 5’-GTCTAGGCCTTAGTTAATAATGAATGC-3’
	Reverse: 5’-CACAGACCTCCTACACTGATATAAACTAT-3’
rs266729	Forward1: 5’-GAACCGGCTCAGATCCTGCC-3
	Forward2: 5’-GAACCGGCTCAGATCCTGCG-3’
	Reverse: 5’-GGACTTTCTTGGCACGCTCATGTTT-3’

### Statistical analysis

Logistic regression and stratified analysis were applied to investigate the different effects of gene and environmental factors on hypertension risk. The Hardy–Weinberg Equilibrium (HWE) of the genotype distributions of rs2241766, rs1501299, and rs266729 in the control group was examined using Chi square (*χ^2^*) goodness-of-fit test through online software SNPStats (*http*:*//bioinfo*.*iconcologia*.*net/SNPstats*). Odds ratios (*ORs*) with 95% confidence intervals (*95%CI*s) for the three SNPs on the hypertension risk were adjusted according to the confounding factors selected by logistic regression. Differences in lipid level between groups were analyzed using t-test. For multiple testing, a powerful bootstrapping method was applied to reduce the potential spurious findings [24].

Crossover analysis was performed to evaluate the effect of gene–lipid interaction on hypertension risk. Lipid levels (TC, LDL-C, HDL-C, and TG) were divided into dichotomized variables using the method above, and rs2241766, rs1501299, and rs266729 were analyzed under the dominant model. A dummy variable was obtained for the four categories by crossing two dichotomized variables: two for the presence of each factor alone (*OR*_10_ or *OR*_01_), one for the presence of both factors (*OR*_11_), and one for the absence of both factors (*OR*_00_) which was used as the reference in the regression model. The *OR* for multiplicative interaction was calculated by *OR*_multi_ = *OR*_11_/*OR*_10_×*OR*_01_. If the interaction between *ADIPOQ* polymorphism and lipid level on hypertension risk was significant, stratified analysis according to lipid level would be conducted. For multiple testing, a powerful bootstrapping method was applied to reduce the potential spurious findings [24].

Significance level was set as *P* < 0.05. Except for HWE and sampling, all other statistical analyses were performed by SPSS 24.0 (IBM Incorporated, New York, United States).

## Results

### Characteristics of participants

A total of 592 participants (296 patients with hypertension and 296 healthy controls) were included, and their characteristics are shown in Table 2. Among them, the minimum and maximum ages were 25 and 60 years, respectively. The cases and controls showed no difference in age (44.20±8.39 vs. 44.40±8.46, *P* = 0.768) and consisted of 257 (86.82%) males and 39 (13.18%) females. A total of 376 (63.51%) subjects worked underground and 216 (36.49%) worked on the ground. Individuals with long work duration (≥16 years), family history of hypertension, alcohol-drinking habit, and high BMI (≥24) were likely susceptible to hypertension (work duration: *OR* = 2.351, *95%CI* = 1.657–3.336; family history of hypertension: *OR* = 1.740, *95%CI* = 1.195–2.535; alcohol-drinking habit: *OR* = 1.546, *95%CI* = 1.083–2.207; BMI: *OR* = 1.879, *95%CI* = 1.312–2.692). The distributions of age, gender, work place, marital status, education, work category, and smoking habit did not differ between the groups.

**Table 2:** *OR*s and *95%CI*s of main risk factors for hypertension.

Variable	Overall		Underground		Ground	
	Ca/Co	*OR* (*95%CI*)	Ca/Co	*OR* (*95%CI*)	Ca/Co	*OR* (*95%CI*)
Gender						
Female	39/39	——	0/0	——	39/39	——
Male	257/257	——	188/188	——	69/69	——
Work place						
Underground	188/188	——	188/188	——	0/0	——
Ground	108/108	——	0/0	——	108/108	——
Marital status						
Married	282/287	1.000	180/183	1.000	102/104	1.000
Others	14/9	1.947(0.796–4.764)	8/5	2.217(0.678–7.249)	6/4	1.766(0.431–7.242)
Education						
Senior high school or above	211/200	1.000	125/123	1.000	86/77	1.000
Junior high school or below	85/96	0.807(0.548–1.189)	63/65	0.882(0.546–1.425)	22/31	0.542(0.265–1.109)
Work category						
Light manual and mental	79/85	1.000	72/84	1.000	7/1	1.000
Heavy manual	217/211	1.097(0.742–1.622)	116/104	1.417(0.907–2.215)	101/107	**0.090(0.010–0.815)**
Work duration (years)						
<16	119/173	1.000	81/120	1.000	38/53	1.000
≥16	177/123	**2.351(1.657–3.336)**	107/68	**2.694(1.721–4.219)**	70/55	**2.157(1.171–3.973)**
Family history of hypertension						
No	190/227	1.000	121/150	1.000	69/77	1.000
Yes	106/69	**1.740(1.195–2.535)**	67/38	**2.101(1.290–3.423)**	39/31	1.461(0.793–2.693)
Alcohol-drinking habit						
No	149/181	1.000	79/98	1.000	70/83	1.000
Yes	147/115	**1.546(1.083–2.207)**	109/90	1.450(0.938–2.239)	38/25	1.551(0.787–3.058)
Smoking habit						
No	118/119	1.000	55/58	1.000	63/61	1.000
Yes	178/177	1.065(0.738–1.538)	133/130	1.131(0.697–1.836)	45/47	0.878(0.471–1.636)
BMI						
<24	85/130	1.000	59/83	1.000	26/47	1.000
≥24	211/166	**1.879(1.312–2.692)**	129/105	1.556(0.989–2.446)	82/61	**2.552(1.365–4.769)**

Ca/Co: Cases and controls.

Stratified analysis was applied to further investigate the difference of environment–factor effects for the participants working underground and on the ground. In the underground group, the work duration and family history of hypertension exhibited significant differences between the cases and controls (work duration: *OR* = 2.694, *95%CI* = 1.721–4.219; family history of hypertension: *OR* = 2.101, *95%CI* = 1.290–3.423). In the ground group, the work category, work duration, and BMI were significantly associated with hypertension (work category: *OR* = 0.090, *95%CI* = 0.010–0.815; work duration: *OR* = 2.157, *95%CI* = 1.171–3.973; BMI: *OR* = 2.552, *95%CI* = 1.365–4.769).

### Genotypes

The genotype distributions of the candidate variants and the associations between the genotype and hypertension risk are shown in Table 3. In the overall control group, the MAFs of rs2241766, rs1501299, and rs266729 were 29.22%, 26.52%, and 25.84%, respectively. The genotype distributions of the three SNPs among the controls were in HWE (rs2241766: *χ^2^* = 3.112, *P* = 0.078; rs1501299: *χ^2^* = 3.012, *P* = 0.083; rs266729: *χ^2^* = 0.457, *P* = 0.499). The association of hypertension risk with rs2241766 and rs1501299 was close to 1, and that with rs266729 was greater than 1 (rs2241766: *OR* = 0.906, *95%CI* = 0.645–1.270; rs1501299: *OR* = 0.902, *95%CI* = 0.643–1.266; rs266729: *OR* = 1.363, *95%CI* = 0.971–1.913).

**Table 3:** Association of hypertension risk with rs2241766, rs1501299, and rs266729.

Variable	Overall			Underground			Ground		
	Ca/Co	*OR* (*95%CI*) ^a^	*P*_Boot_	Ca/Co	*OR* (*95%CI*) ^b^	*P*_Boot_	Ca/Co	*OR* (*95%CI*) ^c^	*P*_Boot_
rs2241766									
TT	153/142	1.000		89/88	1.000		64/54	1.000	
TG	123/135	0.901(0.634–1.280)	0.568	89/87	1.009(0.654–1.556)	0.969	34/48	0.662(0.365–1.203)	0.178
GG	20/19	0.936(0.468–1.872)	0.859	10/13	0.776(0.314–1.920)	0.616	10/6	1.268(0.412–3.905)	0.663
TG+GG	143/154	0.906(0.645–1.270)	0.558	99/100	0.978(0.642–1.490)	0.916	44/54	0.737(0.419–1.297)	0.305
rs1501299									
GG	161/154	1.000		103/93	1.000		58/61	1.000	
GT	116/127	0.876(0.616–1.247)	0.473	72/88	0.646(0.415–1.006)	0.053	44/39	1.532(0.838–2.802)	0.157
TT	19/15	1.100(0.523–2.313)	0.799	13/7	2.036(0.750–5.529)	0.163	6/8	0.769(0.235–2.511)	0.665
GT+TT	135/142	0.902(0.643–1.266)	0.546	85/95	0.738(0.483–1.128)	0.162	50/47	1.380(0.779–2.444)	0.287
rs266729									
CC	147/165	1.000		98/107	1.000		49/58	1.000	
CG	127/109	1.383(0.969–1.973)	0.082	80/67	1.357(0.871–2.114)	0.195	47/42	1.369(0.756–2.479)	0.311
GG	22/22	1.261(0.650–2.445)	0.457	10/14	0.770(0.319–1.862)	0.561	12/8	2.072(0.728–5.901)	0.150
CG+GG	149/131	1.363(0.971–1.913)	0.707	90/81	1.250(0.819–1.909)	0.291	59/50	1.469(0.833–2.589)	0.193

Ca/Co: Cases and controls.

^a^ Adjusted by confounding factors, including work duration, family history of hypertension, alcohol-drinking habit, and BMI.

^b^ Adjusted by confounding factors, including work duration and family history of hypertension.

^c^ Adjusted by confounding factors, including work category, work duration and BMI.

### Lipid levels

TC, LDL-C, HDL-C, and TG levels and their associations with hypertension risk are shown in Table 4. In the overall group, the mean (standard deviation) levels of TC, LDL-C, HDL-C, and TG were 5.17 (0.88), 3.31 (0.75), 1.20 (0.36), and 2.12 (1.82) mmol/L, respectively, for the cases, and 4.84 (0.93), 3.05 (0.70), 1.11 (0.41), and 2.03 (1.75) mmol/L, respectively, for the controls. Significant differences between the cases and controls were found for TC, LDL-C, and HDL-C levels (TC: *t* = -4.546, *P*<0.001; LDL-C: *t* = -4.418, *P*<0.001; HDL-C: *t* = -2.773, *P* = 0.006) but not for TG (*t* = -0.605, *P* = 0.545). In the overall and underground groups, the individuals with high TC (≥5.18 mmol/L), LDL-C (≥3.12 mmol/L), and HDL-C (≥1.04 mmol/L) level were likely susceptible to hypertension (overall: TC: *OR* = 1.807, *95%CI* = 1.266–2.581; LDL-C: *OR* = 1.981, *95%CI* = 1.400–2.803; HDL-C: *OR* = 1.559, *95%CI* = 1.093–2.223; underground: TC: *OR* = 2.274, *95%CI* = 1.446–3.574; LDL-C: *OR* = 2.599, *95%CI* = 1.688–4.003; HDL-C: *OR* = 1.617, *95%CI* = 1.047–2.497). In the ground group, no significant association was found between lipid levels and hypertension risk (all *P*>0.05). The distribution of TG level did not differ between the cases and controls (all *P*>0.05).

**Table 4:** Association of hypertension risk with TC, LDL-C, HDL-C, and TG.

Variable	Overall			Underground			Ground		
	Ca/Co	*OR* (*95%CI*)	*P*_Boot_	Ca/Co	*OR* (*95%CI*)	*P*_Boot_	Ca/Co	*OR* (*95%CI*)	*P*_Boot_
TC									
Low	166/212	1.000		104/139	1.000		62/73	1.000	
High	130/84	**1.807(1.266–2.581)**	**0.001**	84/49	**2.274(1.446–3.574)**	**0.002**	46/35	1.320(0.737–2.366)	0.378
LDL-C									
Low	111/173	1.000		72/117	1.000		39/56	1.000	
High	185/123	**1.981(1.400–2.803)**	**0.002**	116/71	**2.599(1.688–4.003)**	**0.001**	69/52	1.438(0.799–2.588)	0.221
HDL-C									
Low	105/136	1.000		63/89	1.000		42/47	1.000	
High	191/160	**1.559(1.093–2.223)**	**0.019**	125/99	**1.617(1.047–2.497)**	**0.027**	66/61	1.257(0.704–2.245)	0.445
TG									
Low	143/158	1.000	0.738	85/98	1.000	0.268	58/60	1.000	0.767
High	153/138	1.060(0.747–1.504)		103/90	1.293(0.846–1.975)		50/48	0.920(0.519–1.632)	

Ca/Co: Cases and controls.

^a^ Adjusted by confounding factors, including work duration, family history of hypertension, alcohol-drinking habit, and BMI.

^b^ Adjusted by confounding factors, including work duration and family history of hypertension.

^c^ Adjusted by confounding factors, including work category, work duration and BMI.

### Association of gene–lipid interaction with hypertension risk

As shown in Table 5, crossover analysis was performed to evaluate the gene–lipid interaction between *ADIPOQ* polymorphism and lipid levels. On the basis of results in Table 3 and the conclusions from meta-analyses [25, 26], rs2241766 TG+GG, rs1501299 GG, and rs266729 CG+GG were considered as high risk genotypes. For a dummy variable, the category with low risk genotype and low lipid level was used as reference. In the overall and underground groups, rs2241766 TG+GG and high TC level increased the hypertension risk (overall: rs2241766, *OR* = 1.279, *95%CI* = 0.835–1.958; TC, *OR* = 2.900, *95%CI* = 1.727–4.871; underground: rs2241766, *OR* = 1.519, *95%CI* = 0.894–2.583; TC, *OR* = 4.758, *95%CI* = 2.346–9.648), whereas the multiplicative interaction of these two factors decreased the hypertension risk (overall: *OR* = 0.393, *95%CI* = 0.191–0.806; underground: *OR* = 0.264, *95%CI* = 0.104–0.670). Similarly, the multiplicative interaction among rs2241766, rs1501299, and lipid levels reduced the hypertension risk in the overall and underground groups (rs1501299 and TC: overall, *OR* = 0.445, *95%CI* = 0.216–0.918, underground, *OR* = 0.460, *95%CI* = 0.185–1.143; rs2241766 and LDL-C: overall, *OR* = 0.440, *95%CI* = 0.221–0.877, underground, *OR* = 0.291, *95%CI* = 0.121–0.701; rs1501299 and HDL-C: overall, *OR* = 0.479, *95%CI* = 0.237–0.967, underground, *OR* = 0.392, *95%CI* = 0.162–0.951). The multiplicative interactions for other *ADIPOQ* polymorphisms and lipid levels were not significant (*95%CIs* included 1 and *P*_BOOT_ > 0.05).

**Table 5:** Effects of rs2241766, rs1501299, rs266729 interaction with lipid levels on hypertension risk.

Genotype	Lipid level	Overall			Underground			Ground		
		Ca/Co	*OR* (*95%CI*) ^a^	*P*_Boot_	Ca/Co	*OR* (*95%CI*) ^b^	*P*_Boot_	Ca/Co	*OR* (*95%CI*) ^c^	*P*_Boot_
rs2241766	TC									
TT	Low	75/108	1.000		42/72	1.000		33/36	1.000	
TG+GG	Low	91/104	1.279(0.835–1.958)	0.245	62/67	1.519(0.894–2.583)	0.125	29/37	0.845(0.416–1.716)	0.644
TT	High	78/34	**2.900(1.727–4.871)**	**0.001**	47/16	**4.758(2.346–9.648)**	**0.001**	31/18	1.479(0.676–3.238)	0.341
TG+GG	High	52/50	1.457(0.876–2.422)	0.153	37/33	**1.912(1.022–3.576)**	**0.045**	15/17	0.907(0.373–2.201)	0.838
Multiplicative interaction			**0.393(0.191–0.806)**	**0.011**		**0.264(0.104–0.670)**	**0.002**		0.725(0.221–2.375)	0.602
rs1501299	TC									
GT+TT	Low	70/110	1.000		41/73	1.000		51/38	1.000	
GG	Low	96/102	1.461(0.952–2.244)	0.097	63/66	**1.816(1.063–3.101)**	**0.028**	50/36	0.884(0.430–1.820)	0.737
GT+TT	High	65/32	**2.837(1.644–4.894)**	**0.001**	44/22	**3.453(1.774–6.723)**	**0.001**	30/11	2.001(0.780–5.134)	0.165
GG	High	65/52	**1.844(1.126–3.019)**	**0.019**	40/27	**2.886(1.509–5.520)**	**0.002**	41/25	0.937(0.429–2.048)	0.870
Multiplicative interaction			**0.445(0.216–0.918)**	**0.035**		0.460(0.185–1.143)	0.087		0.529(0.158–1.778)	0.324
rs266729	TC									
CC	Low	86/114	1.000		55/74	1.000		31/40	1.000	
CG+GG	Low	80/98	1.212(0.790–1.858)	0.381	49/65	1.142(0.673–1.937)	0.625	31/33	1.457(0.703–3.017)	0.318
CC	High	61/51	1.553(0.954–2.528)	0.072	43/33	**1.944(1.070–3.532)**	**0.037**	18/18	1.302(0.564–3.006)	0.547
CG+GG	High	69/33	**2.620(1.558–4.406)**	**0.002**	41/16	**3.358(1.667–6.763)**	**0.004**	28/17	1.864(0.833–4.173)	0.125
Multiplicative interaction			1.393(0.675–2.873)	0.401		1.513(0.597–3.836)	0.383		0.983(0.300–3.220)	0.980
rs2241766	LDL-C									
TT	Low	46/89	1.000		26/61	1.000		20/28	1.000	
TG+GG	Low	65/84	1.408(0.856–2.316)	0.158	46/56	1.827(0.984–3.395)	0.056	19/28	0.771(0.327–1.818)	0.544
TT	High	107/53	**3.036(1.830–5.034)**	**0.001**	63/27	**5.120(2.638–9.936)**	**0.001**	44/26	1.455(0.644–3.283)	0.374
TG+GG	High	78/70	**1.882(1.144–3.096)**	**0.009**	53/44	**2.722(1.454–5.097)**	**0.003**	25/26	1.064(0.461–2.455)	0.898
Multiplicative interaction			**0.440(0.221–0.877)**	**0.021**		**0.291(0.121–0.701)**	**0.006**		0.948(0.297–3.030)	0.918
rs1501299	LDL-C									
GT+TT	Low	47/83	1.000		29/58	1.000		18/25	1.000	
GG	Low	64/90	1.213(0.736–2.000)	0.452	43/59	1.624(0.875–3.015)	0.134	21/31	0.711(0.296–1.705)	0.457
GT+TT	High	88/59	**2.133(1.280–3.555)**	**0.005**	56/37	**3.039(1.610–5.737)**	**0.002**	32/22	1.376(0.572–3.306)	0.478
GG	High	97/64	**2.274(1.378–3.753)**	**0.001**	60/34	**3.804(2.006–7.212)**	**0.001**	37/30	1.035(0.442–2.423)	0.944
Multiplicative interaction			0.879(0.442–1.749)	0.697		0.770(0.322–1.841)	0.573		1.059(0.334–3.355)	0.913
rs266729	LDL-C									
CC	Low	59/93	1.000		37/62	1.000		22/31	1.000	
CG+GG	Low	52/80	1.083(0.659–1.780)	0.747	35/55	1.150(0.626–2.111)	0.651	17/25	1.196(0.499–2.869)	0.690
CC	High	88/72	**1.618(1.008–2.597)**	**0.036**	61/45	**2.317(1.295–4.148)**	**0.002**	27/27	1.199(0.537–2.678)	0.667
CG+GG	High	97/51	**2.674(1.640–4.361)**	**0.001**	55/26	**3.579(1.881–6.809)**	**0.001**	42/25	1.927(0.886–4.188)	0.082
Multiplicative interaction			1.526(0.765–3.043)	0.237		1.343(0.560–3.224)	0.672		1.343(0.423–4.263)	0.619
rs2241766	HDL-C									
TT	Low	46/63	1.000		25/43	1.000		21/20	1.000	
TG+GG	Low	59/73	1.125(0.659–1.918)	0.669	38/46	1.399(0.711–2.752)	0.348	21/27	0.912(0.382–2.179)	0.840
TT	High	107/79	**1.811(1.090–3.008)**	**0.028**	64/45	**2.199(1.149–4.209)**	**0.021**	43/34	1.402(0.629–3.125)	0.403
TG+GG	High	84/81	1.509(0.895–2.545)	0.130	61/54	1.755(0.926–3.325)	0.089	23/27	0.924(0.382–2.233)	0.865
Multiplicative interaction			0.741(0.371–1.479)	0.418		0.570(0.239–1.363)	0.340		0.722(0.227–2.292)	0.570
rs1501299	HDL-C									
GT+TT	Low	38/67	1.000		23/47	1.000		15/20	1.000	
GG	Low	67/69	**1.765(1.023–3.044)**	**0.035**	40/42	**2.435(1.215–4.879)**	**0.011**	27/27	1.075(0.440–2.622)	0.849
GT+TT	High	97/75	**2.355(1.386–4.001)**	**0.003**	62/48	**2.710(1.406–5.222)**	**0.002**	35/27	1.748(0.728–4.198)	0.214
GG	High	94/85	**1.991(1.179–3.363)**	**0.009**	63/51	**2.586(1.351–4.949)**	**0.004**	31/34	0.990(0.412–2.377)	0.979
Multiplicative interaction			**0.479(0.237–0.967)**	**0.031**		**0.392(0.162–0.951)**	**0.008**		0.527(0.165–1.681)	0.260
rs266729	HDL-C									
CC	Low	50/80	1.000		32/53	1.000		18/27	1.000	
CG+GG	Low	55/56	1.648(0.965–2.815)	0.064	31/36	1.614(0.821–3.173)	0.163	24/20	1.770(0.735–4.262)	0.166
CC	High	97/85	**1.813(1.117–2.942)**	**0.021**	66/54	**1.968(1.089–3.556)**	**0.019**	31/31	1.464(0.646–3.317)	0.374
CG+GG	High	94/75	**2.138(1.306–3.500)**	**0.005**	59/45	**2.038(1.110–3.741)**	**0.024**	35/30	1.858(0.827–4.175)	0.138
Multiplicative interaction			0.716(0.357–1.435)	0.330		0.642(0.268–1.537)	0.304		0.717(0.228–2.252)	0.561
rs2241766	TG									
TT	Low	69/76	1.000		37/45	1.000		32/31	1.000	
TG+GG	Low	74/82	1.028(0.639–1.653)	0.907	48/53	1.094(0.596–2.008)	0.767	26/29	0.937(0.437–2.009)	0.857
TT	High	84/66	1.205(0.738–1.967)	0.462	52/43	1.432(0.772–2.658)	0.274	32/23	1.169(0.540–2.534)	0.690
TG+GG	High	69/72	0.960(0.588–1.567)	0.858	51/47	1.291(0.699–2.383)	0.401	18/25	0.641(0.282–1.460)	0.268
Multiplicative interaction			0.775(0.395–1.523)	0.447		0.824(0.354–1.918)	0.662		0.585(0.187–1.826)	0.384
rs1501299	TG									
GT+TT	Low	68/82	1.000		38/53	1.000		30/29	1.000	
GG	Low	75/76	1.114(0.692–1.793)	0.657	47/45	1.613(0.876–2.969)	0.149	28/31	0.717(0.330–1.555)	0.409
GT+TT	High	67/60	1.062(0.639–1.767)	0.820	47/42	1.542(0.833–2.854)	0.164	20/18	0.927(0.393–2.189)	0.863
GG	High	86/78	1.165(0.725–1.871)	0.566	56/48	1.739(0.961–3.146)	0.070	30/30	0.689(0.316–1.501)	0.374
Multiplicative interaction			0.985(0.499–1.941)	0.971		0.699(0.300–1.631)	0.422		1.036(0.326–3.293)	0.957
rs266729	TG									
CC	Low	69/88	1.000		43/55	1.000		26/33	1.000	
CG+GG	Low	74/70	1.454(0.902–2.344)	0.126	42/43	1.228(0.669–2.254)	0.497	32/27	1.584(0.814–3.082)	0.173
CC	High	78/77	1.129(0.704–1.812)	0.634	55/52	1.272(0.715–2.264)	0.426	23/25	1.122(0.561–2.245)	0.750
CG+GG	High	75/61	1.442(0.883–2.356)	0.121	48/38	1.636(0.891–3.006)	0.116	27/23	1.104(0.539–2.261)	0.776
Multiplicative interaction			0.877(0.446–1.728)	0.707		1.047(0.448–2.451)	0.921		0.621(0.228–1.688)	0.351

Ca/Co: Cases and controls.

^a^ Adjusted by confounding factors, including work duration, family history of hypertension, alcohol-drinking habit, and BMI.

^b^ Adjusted by confounding factors, including work duration and family history of hypertension.

^c^ Adjusted by confounding factors, including work category, work duration and BMI.

### Association of hypertension risk with rs2241766 and rs1501299 at two lipid levels

Given the significant interaction between *ADIPOQ* polymorphism and lipid levels, stratified analysis by lipid level was further conducted (Table 6). In the overall and underground groups, the hypertension risk was high for the subjects with rs2241766 TG+GG under the low lipid level (TC: overall, *OR* = 1.332, *95%CI* = 0.861–2.060, underground, *OR* = 1.514, *95%CI* = 0.889–2.577; LDL-C: overall, *OR* = 1.419, *95%CI* = 0.854–2.359, underground, *OR* = 1.824, *95%CI* = 0.977–3.403) and low for those under the high lipid level (TC: overall, *OR* = 0.458, *95%CI* = 0.256–0.818, underground, *OR* = 0.397, *95%CI* = 0.185–0.851; LDL-C: overall, *OR* = 0.583, *95%CI* = 0.361–0.943, underground, *OR* = 0.528, *95%CI* = 0.282–0.988) with rs2241766 TT as the reference. Compared with the GT+TT genotype, the GG genotype for rs1501299 increased the hypertension risk under low lipid level (TC: overall, *OR* = 1.466, *95%CI* = 0.946–2.272, underground, *OR* = 1.819 *95%CI* = 1.061–3.116; HDL-C: overall, *OR* = 1.838, *95%CI* = 1.029–3.282, underground, *OR* = 2.647 *95%CI* = 1.257–5.577) and decreased the hypertension risk at the high lipid level (TC: overall, *OR* = 0.687, *95%CI* = 0.386–1.224, underground, *OR* = 0.843, *95%CI* = 0.403–1.763; HDL-C: overall, *OR* = 0.866, *95%CI* = 0.560–1.337, underground, *OR* = 0.962, *95%CI* = 0.559–1.654). In the ground group, hypertension risk was not significantly associated with rs2241766 and rs1501299 at both lipid levels (*95%CIs* included 1 and *P* > 0.05).

**Table 6:** Associations of hypertension risk with rs2241766 and rs150129 stratified by lipid level.

Variable	TC level	TT/GT+TT		TG+GG/GG		
		Ca/Co	*OR* (*95%CI*)	Ca/Co	*OR* (*95%CI*)	*P*_Boot_
rs2241766	TC					
Overall ^a^	Low	75/108	1.000	91/104	1.332(0.861–2.060)	0.181
	High	78/34	1.000	52/50	**0.458(0.256–0.818)**	**0.009**
Underground ^b^	Low	42/72	1.000	62/67	1.514(0.889–2.577)	0.121
	High	31/18	1.000	15/17	**0.397(0.185–0.851)**	**0.027**
Ground ^c^	Low	33/36	1.000	29/37	0.842(0.409–1.731)	0.661
	High	48/19	1.000	23/17	0.537(0.203–1.420)	0.214
rs1501299	TC					
Overall ^a^	Low	97/121	1.000	122/108	1.466(0.946–2.272)	0.099
	High	65/32	1.000	65/52	0.687(0.386–1.224)	0.205
Underground ^b^	Low	41/73	1.000	63/66	**1.819(1.061–3.116)**	**0.038**
	High	44/22	1.000	40/7	0.843(0.403–1.763)	0.663
Ground ^c^	Low	21/10	1.000	25/25	0.831(0.396–1.746)	0.629
	High	30/11	1.000	41/25	0.437(0.166–1.150)	0.099
rs2241766	LDL-C					
Overall ^a^	Low	46/89	1.000	65/84	1.419(0.854–2.359)	0.179
	High	107/53	1.000	78/70	**0.583(0.361–0.943)**	**0.042**
Underground ^b^	Low	26/61	1.000	46/56	1.824(0.977–3.403)	0.063
	High	63/27	1.000	53/44	**0.528(0.282–0.988)**	**0.045**
Ground ^c^	Low	20/28	1.000	19/28	0.714(0.294–1.732)	0.466
	High	44/26	1.000	25/26	0.686(0.316–1.491)	0.357
rs1501299	HDL-C					
Overall ^a^	Low	67/69	1.000	38/67	**1.838(1.029–3.282)**	**0.041**
	High	94/85	1.000	97/75	0.866(0.560–1.337)	0.537
Underground ^b^	Low	40/42	1.000	23/47	**2.647(1.257–5.577)**	**0.016**
	High	63/51	1.000	62/48	0.962(0.559–1.654)	0.878
Ground ^c^	Low	27/27	1.000	15/20	1.022(0.405–2.582)	0.959
	High	31/34	1.000	35/27	0.604(0.286–1.276)	0.189

Ca/Co: Cases and controls.

^a^ Adjusted by confounding factors, including work duration, family history of hypertension, alcohol-drinking habit, and BMI.

^b^ Adjusted by confounding factors, including work duration and family history of hypertension.

^c^ Adjusted by confounding factors, including work category, work duration and BMI.

### Lipid level in rs2241766 and rs1501299 genotypes

The lipid levels in rs2241766 and rs1501299 genotypes were compared in the case and control groups (Table 7). In the case group, the lipid levels for rs2241766 TG+GG were lower than those for rs2241766 TT (TC: *t* = 2.500, *P* = 0.013; LDL-C: *t* = 1.850, *P* = 0.065). Meanwhile, the lipid levels for rs1501299 GT+TT were higher than those for rs1501299 GG (TC: *t* = -2.859, *P* = 0.005; HDL-C: *t* = -3.408, *P* = 0.001). In the control group, the lipid levels in rs2241766 and rs1501299 genotypes were not significant (all *P* > 0.05).

**Table 7:** Lipid levels in rs2241766 and rs1501299 genotypes.

Variable	Case		Control	
	$\left(\bar{x}\pm S\right)$	*t/P* ^*^	$\left(\bar{x}\pm S\right)$	*t/P* ^*^
rs2241766	TC		TC	
TT	5.296±0.829	**2.500/0.013**	4.764±0.894	-1.295/0.197
TG+GG	5.043±0.913		4.903±0.954	
rs1501299	TC		TC	
GG	5.042±0.801	**-2.859/0.005**	4.884±0.980	0.925/0.356
GT+TT	5.331±0.941		4.784±0.866	
rs2241766	LDL-C		LDL-C	
TT	3.385±0.732	1.850/0.065	2.986±0.742	-1.404/0.161
TG+GG	3.225±0.758		3.100±0.651	
rs1501299	HDL-C		HDL-C	
GG	1.135±0.325	**-3.408/0.001**	1.132±0.423	0.890/0.370
GT+TT	1.276±0.388		1.089±0.391	

* T-test for the wild homozygous group versus the heterozygous+mutant homozygous group.

## Discussion

In this case-control study, we explored the association among *ADIPOQ* polymorphisms, serum lipid levels, and hypertension risk in coal miners. Results showed that *ADIPOQ* polymorphisms alone were not associated with hypertension. With the increase in TC, LDL-C, and HDL-C levels, the risk of hypertension also increased. Further interaction analysis revealed antagonistic interactions between *ADIPOQ* polymorphisms and lipid levels.

In this study, we found no significant relationship between *ADIPOQ* polymorphisms and hypertension risk in coal miners. The negative results may be attributed to the limited study power and the healthy worker effect on coal miners. According to systematic reviews and meta-analyses those pooled and expanded the sample size, hypertension risk is increased by rs2241766 TG+GG and rs266729 CG+GG but decreased by rs1501299 GT+TT [25, 26]. This finding is similar to the present conclusion. *ADIPOQ* located on chromosome 3q27 is a susceptibility locus for metabolic syndrome and is composed of three exons and two introns spanning a 17 kb region [27]. *ADIPOQ* rs2241766, rs1501299, and rs266729 have been examined in several studies, and the results indicate that rs2241766 TG+GG, rs1501299 GG, and rs266729 CG+GG are associated with decreased adiponectin level and increased insulin resistance [12, 13]. Adiponectin is an important adipocyte-derived plasma protein and is highly abundant in blood. In contrast to other adipocytokines, adiponectin is significantly negatively associated with insulin resistance, diabetes, and hypertension [7–9]. In adiponectin knock-out mice, the intravenous and intracerebroventricular injection of adiponectin decreased renal sympathetic nervous system activity and blood pressure [28]. Wildman et al. showed that a 1 ln μg/mL decline in adiponectin levels over 10 years was associated with 12.3 mmHg increase in systolic blood pressure [29]. A systematic review by Kim et al. showed that hypertensive adults had lower mean adiponectin levels than normotensive adults, and an inverse monotonic relationship occurs between adiponectin levels and the future risk of hypertension [10]. Hypoadiponectinemia could cause hypertension through several potential mechanisms, such as endothelial dysfunction, insulin resistance, sympathetic activation, increased circulating fatty acid levels via reduced fatty acid oxidation, impaired endothelium-dependent vasodilation, and vascular inflammation [30]. Thus, the effects of adiponectin and *ADIPOQ* polymorphism on hypertension risk are biologically plausible.

The China National Diabetes and Metabolic Disorders Study showed that the mean levels of TC, LDL-C, HDL-C, and TG were 4.72, 2.68, 1.30, and 1.57 mmol/L, respectively [31]. The corresponding average levels of TC, LDL-C, and TG in the present work were relatively high: 5.17 (0.88), 3.31 (0.75), and 2.12 (1.82) mmol/L for cases, respectively, and 4.84 (0.93), 3.05 (0.70), and 2.03 (1.75) mmol/L for controls, respectively. By contrast, the average levels of HDL-C were relatively low: 1.20 (0.36) mmol/L for cases and 1.11(0.41) mmol/L for controls. Owing to their long exposure to coal dust, coal miners are likely to be affected by disorders of lipid metabolism [32] and thus susceptible to hypertension. In the present study, the individuals with high levels of TC (≥5.18 mmol/L), LDL-C (≥3.12 mmol/L), and HDL-C (≥1.04 mmol/L) were likely susceptible to hypertension. Except HDL-C, all lipid levels in the current work were similar to those in previous studies [18–21]. Two large population-based cohort studies from China and Japan indicated that the increased occurrence of hypertension is associated with increased TC and LDL-C and decreased HDL-C [18, 33]. The possible pathophysiological mechanism is that the high blood lipid levels increase blood viscosity, thereby augmenting peripheral resistance; in addition, the loss of physiological vasomotor activity resulting from endothelial damage may finally manifest as hypertension [34]. Endothelial damage is possibly caused by impaired nitric oxide production and activity and alterations in endothelin-1 and endothelin A and B receptor expression for individuals with dyslipidemia [35]. Oxidative stress is promoted by lipid abnormalities, leading to insulin resistance and increased production of renin–angiotensin–aldosterone system components [17]. In turn, these biological changes may increase the blood pressure. Hence, dyslipidemia can promote hypertension risk.

Contrary to most studies, the present work found that coal miners with high HDL-C levels, especially those working underground, were at a great risk of developing hypertension. Only a few reports came to conclusions different from the majority. Zhang et al. [36] and Paynter et al. [37] indicated that hypertensive patients have dramatically low plasma large HDL-C and large HDL-C percentage and high small HDL-C and small HDL-C percentage. These results suggest that the hypertension prediction value of HDL-C subfraction is higher than that of HDL-C. An animal study examining the relationship between subchronic air pollution and obesity reported that chocolate and residual oil fly ash (ROFA)+chocolate groups showed higher levels of HDL-C, TC, and TG than the control and ROFA groups [38]. Chocolate contains cocoa that could increase HDL-C level. Otsuka et al. [18] found a U-shaped relationship between HDL-C level and hypertension risk. Compared with the third quintile, the multi-adjusted hazard ratio in the lowest quintile and the highest quintile were all greater than 1 (*P*<0.05). People with increased circulating HDL-C levels are susceptible to heritable cholesteryl ester transfer protein (CETP) deficiency, which further promotes HDL-C dysfunction. In addition, HDL-C dysfunction impairs functional and structural arterial properties and thus increases hypertension risk. The highest level of HDL-C for the preceding study was 73–162 mg/dL (4.05–9.00 mmol/L), which was above the normal range. The HDL-C levels of coal miners in the current work were 1.20 (0.36) and 1.11 (0.41) mmol/L for the cases and controls, respectively. These values were lower than the average level from the China National Diabetes and Metabolic Disorders Study. Therefore, the above explanation is not applicable to the present subjects. Three possible explanations are offered for the present results. First, the elevated small HDL-C and small HDL-C percentage in the case group might have promoted hypertension. Second, local environment or diet might influence HDL-C level (discussed later). Third, coal miners might have better health and with a lower HDL-C level compared with the general population. When the body is in a state of hyperlipidemia and/or hypertension, HDL-C is activated to play a protective function. Hence, HDL-C levels were elevated in the case group, and the interaction between *ADIPOQ* polymorphisms and lipid levels generated protective effects. The actual mechanism must be further explored.

The interactions between *ADIPOQ* polymorphisms and lipid levels were detected in this study. Crossover analysis results showed antagonistic interactions between rs2241766 and TC, rs1501299 and TC, rs2241766 and LDL-C, and rs1501299 and HDL-C. Although the interaction between *ADIPOQ* polymorphism and lipid levels on hypertension risk has not been explored, numerous studies focused on the relationship between these two factors. Pineda-Tenor D‘s et al. [39] found that subjects with the rs2241766 TG+GG genotype had significantly lower serum levels of TC and LDL-C than rs2241766 TT carriers. A meta-analysis conducted by Zhao et al. [40] in 2011 indicated that rs1501299 GT+TT had low levels of TC. Two studies in 2017 [41] and 2019 [42] showed that the GG genotype of rs1501299 had low levels of TC. A meta-analysis performed by Su et al. [43] proposed that the T allele carriers of rs1501299 polymorphism had higher levels of HDL-C and adiponectin than GG homozygotes, whereas the G allele carriers of rs2241766 polymorphism had lower levels of HDL-C and adiponectin than TT homozygotes. The above results indicated that the associations between *ADIPOQ* polymorphisms and lipid levels remain inconclusive. Adiponectin and dyslipidemia have many common pathways in the pathogenesis of hypertension, such as endothelial dysfunction, insulin resistance, and reduced release of nitric oxide [16–18]. In addition, adiponectin can directly affect lipid levels. A rat experiment showed that adipocytes regulated hepatic cholesterol metabolism partly via adiponectin [14]. Adiponectin and its receptors increased cholesterol efflux at least partially through an ATP binding cassette transporter A1 pathway, suggesting that adiponectin might enhance the reverse cholesterol transport system and induce an anti-atherogenic effect [15]. The synergistic effect of *ADIPOQ* polymorphism and dyslipidemia on hypertension has a biological basis, but the exact molecular mechanism for the antagonistic interactions between the two factors remains unclear.

Further stratified analysis showed that for the subjects with rs2241766 TG+GG or rs1501299 GG in the overall and underground groups, the hypertension risk was high for those under the low lipid level but low for those under the high lipid level. The differences of lipid levels corresponding to *ADIPOQ* genotypes were compared. In the case group, the TC and LDL-C levels for rs2241766 TG+GG were lower than those for rs2241766 GG, and the TC and HDL-C levels for rs1501299 GT+TT were higher than those for rs1501299 GG. These results were consistent with previous studies [18, 40–42]. The low proportion of dietary fiber and unsaturated fatty acid in diet, the activation of peroxisome proliferator-activated receptor-γ by some fatty acids from the diet, and the differences of age, health status, and obesity degree of subjects were the possible explanations for their findings. Especially, diet and the particularity of the research subjects are the main reasons. The antagonistic interactions between *ADIPOQ* polymorphism and dyslipidemia could be explained as follows. First, some special foods in the local diet might have influenced the lipid levels. The Datong Coal Mine, located in the north of Shanxi Province, has a temperate continental climate with cold and dry weather and has a large temperature difference between day and night, which is suitable for the growth of oats. Thus, local residents have a habit of eating oats. Some studies focused on the efficacy of oat intake. Two population-based randomized controlled trials reported a decrease in TC, LDL-C, and blood pressure and an increase in HDL-C in the oat group compared with those in the control group [44, 45]. Two animal studies showed similar results and found a significant increase in fecal bile acids in the oat group, indicating that dietary oat improved hypercholesterolemia by increasing the excretions of fecal bile acids [46, 47]. In addition to oat, people in Datong also prefer to eat buckwheat, millet, corn, and other coarse grains, which may have contributed to improving blood lipid levels and regulating blood pressure. Second, coal miners have better health and lower hypertension risk than the general population under the same situation, and this phenomenon is called the healthy worker effect. Self-protective ability is activated when the body is in a state of hyperlipidemia and/or hypertension. The promoting mechanism may involve lipid metabolism, adiponectin level, and insulin resistance and must be further explored.

Several limitations exist in this study. First, this retrospective work cannot bypass the intrinsic limitation of case-control study design. For result validation, replication in cohorts is needed. Second, instead of a random sample from the general population, the selection of coal miners as subjects might limit the generalizability of the results. Third, the sample size was relatively small and thus might not provide sufficient statistical power to detect the weak genetic effects of *ADIPOQ* polymorphism on hypertension risk. Fourth, the evaluation of serum adiponectin level to confirm the relationship among *ADIPOQ* polymorphism, lipid level, and hypertension risk was not conducted. Fifth, investigation about oat intake was absent, which is important to this study. Thus, prospective studies based on general population with large sample size should be replicated in the future.

## Conclusions

Although the effects of *ADIPOQ* polymorphisms alone were not remarkable, an antagonistic interaction was found between *ADIPOQ* polymorphisms and lipid levels. Oat intake is a possible cause of this phenomenon. The findings must be verified by future studies on local general population with large sample size.

## Supporting information

S1 Checklist STROBE statement—checklist of items that should be included in reports of observational studies. (DOCX)

## References

[1] Global Health Observatory data [Internet]. World Health Organization [cited 2021 Sep 20]. Available from: http://www.who.int/gho/ncd/risk_factors/blood_pressure_prevalence_text/en/.

[2] Zhang M, Wu J, Zhang X, Hu CH, Zhao ZP, Li C, et al. [Prevalence and control of hypertension in adults in China, 2018]. Zhonghua Liu Xing Bing Xue Za Zhi. 2021; 42(10):1780–9. doi: 10.3760/cma.j.cn112338-20210508-00379 . Chinese. 34814612

[3] Ustinova OIu, Alekseev VB, Rumiantseva AN, IaV Orehova. [Influence of work intensity on development of arterial hypertension in metal-mining workers]. Med Tr Prom Ekol. 2013; (11):27–31. . Russian. 24640088

[4] Alif SM, Sim MR, Ho C, Glass DC. Cancer and mortality in coal mine workers: a systematic review and meta-analysis. Occup Environ Med. 2022 May; 79(5): 347–57. doi: 10.1136/oemed-2021-107498 34782367

[5] Schindler M, Pendzialek M, Grybel KJ, Seeling T, Gürke J, Fischer B, et al. Adiponectin stimulates lipid metabolism via AMPK in rabbit blastocysts. Hum Reprod. 2017 Jul 1; 32(7):1382–92. doi: 10.1093/humrep/dex087 28472298 PMC5850832

[6] Ding Y, Zhang D, Wang B, Zhang Y, Wang L, Chen X, et al. APPL1- mediated activation of STAT3 contributes to inhibitory effect of adiponectin on hepatic gluconeogenesis. Mol Cell Endocrinol. 2016 Sep 15;433:12–9. doi: 10.1016/j.mce.2016.05.021 27246173

[7] Liu W, Zhou X, Li Y, Zhang S, Cai X, Zhang R, et al. Serum leptin, resistin, and adiponectin levels in obese and non-obese patients with newly diagnosed type 2 diabetes mellitus: A population-based study. Medicine (Baltimore). 2020 Feb; 99(6):e19052. doi: 10.1097/MD.0000000000019052 32028423 PMC7015632

[8] Daimon M, Oizumi T, Kato T. [Decreased serum levels of adiponectin as a risk for development of type 2 diabetes, and impaired glucose tolerance as a risk for stroke—the Funagata study]. Nihon Rinsho. 2012 May; 70(suppl 3):256–9. . Japanese. 22768529

[9] Drivsholm A, Lund MAV, Hedley PL, Jespersen T, Christiansen M, Hansen T, et al. Associations between thyroid-stimulating hormone, blood pressure and adiponectin are attenuated in children and adolescents with overweight or obesity. J Pediatr Endocrinol Metab. 2019 Dec 18; 32(12):1351–8. doi: 10.1515/jpem-2019-0359 31714888

[10] Kim DH, Kim C, Ding EL, Townsend MK, Lipsitz LA. Adiponectin levels and the risk of hypertension: a systematic review and meta-analysis. https://www.ncbi.nlm.nih.gov/pubmed/?term=FuruhashiM[Author]&cauthor=true&cauthor_uid=16025741Hypertension. 2013 Jul; 62(1):27–32. 10.1161/HYPERTENSIONAHA.113.01453 23716587 PMC3729220

[11] Rojas E, Rodríguez-Molina D, Bolli P, Israili ZH, Faría J, Fidilio E, et al. The role of adiponectin in endothelial dysfunction and hypertension. Curr Hypertens Rep. 2014 Aug; 16(8):463. doi: 10.1007/s11906-014-0463-7 24924994

[12] Chung HK, Chae JS, Hyun YJ, Paik JK, Kim JY, Jang Y, et al. Influence of adiponectin gene polymorphisms on adiponectin level and insulin resistance index in response to dietary intervention in overweight-obese patients with impaired fasting glucose or newly diagnosed type 2 diabetes. Diabetes Care. 2009 Apr; 32(4):552–8. doi: 10.2337/dc08-1605 19131459 PMC2660454

[13] de Luis DA, Calvo SG, Pacheco D, Ovalle HF, Aller R. Adiponectin gene variant RS rs266729: Relation to lipid profile changes and circulating adiponectin after bariatric surgery. Surg Obes Relat Dis. 2018 Sep; 14(9):1402–8. doi: 10.1016/j.soard.2018.06.006 30037702

[14] Li Y, Qin G, Liu J, Mao L, Zhang Z, Shang J. Adipose tissue regulates hepatic cholesterol metabolism via adiponectin. Life Sci. 2014 Nov 18; 118(1):27–33. doi: 10.1016/j.lfs.2014.10.003 25445438

[15] Kitajima K, Miura S, Yamauchi T, Uehara Y, Kiya Y, Rye KA, et al. Possibility of increasing cholesterol efflux by adiponectin and its receptors through the ATP binding cassette transporter A1 in HEK293T cells. Biochem Biophys Res Commun. 2011 Jul 29; 411(2):305–11. doi: 10.1016/j.bbrc.2011.06.131 21740892

[16] Taverne F, Richard C, Couture P, Lamarche B. Abdominal obesity, insulin resistance, metabolic syndrome and cholesterol homeostasis. Pharm Nutr. 2013 October; 1(4):130–6. 10.1016/j.phanu.2013.07.003

[17] Manrique C, Lastra G, Gardner M, Sowers JR. The renin angiotensin aldosterone system in hypertension: roles of insulin resistance and oxidative stress. Med Clin North Am. 2009 May; 93(3):569–82. doi: 10.1016/j.mcna.2009.02.014 19427492 PMC2828938

[18] Otsuka T, Takada H, Nishiyama Y, Kodani E, Saiki Y, Kato K, et al. Dyslipidemia and the Risk of Developing Hypertension in a Working-Age Male Population. J Am Heart Assoc. 2016 Mar 25; 5(3):e003053. doi: 10.1161/JAHA.115.003053 27016576 PMC4943276

[19] Guo ZR, Hu XS, Wu M, Zhou MH, Zhou ZY. A prospective study on the association between dyslipidemia and hypertension. Zhonghua Liu Xing Bing Xue Za Zhi. 2009 Jun; 30(6):554–8. . Chinese. 19957617

[20] Tohidi M, Hatami M, Hadaegh F, Azizi F. Triglycerides and triglycerides to high-density lipoprotein cholesterol ratio are strong predictors of incident hypertension in Middle Eastern women. J Hum Hypertens. 2012 Sep; 26(9):525–32. doi: 10.1038/jhh.2011.70 21776016

[21] Laaksonen DE, Niskanen L, Nyyssönen K, Lakka TA, Laukkanen JA, Salonen JT. Dyslipidaemia as a predictor of hypertension in middle-aged men. Eur Heart J. 2008 Oct; 29(20):2561–8. doi: 10.1093/eurheartj/ehn061 18308688 PMC2721716

[22] Liu LS, Writing Group of 2010 Chinese Guidelines for the Management of Hypertension. [2010 Chinese guidelines for the management of hypertension]. Zhonghua Xin Xue Guan Bing Za Zhi. 2011 Jul; 39(7):579–615. 10.3760/cma.j.issn.0253-3758.2011.07.002 . Chinese. 22088239

[23] Joint committee for guideline revision. 2016 Chinese guideline for the management of dyslipidemia in adults. J Geriatr Cardiol. 2018 Jan;15(1):1–29. doi: 10.11909/j.issn.1671-5411.2018.01.011 29434622 PMC5803534

[24] Andersson T, Alfredsson L, Källberg H, Zdravkovic S, Ahlbom A. Calculating measures of biological interaction. Eur J Epidemiol. 2005; 20(7):575–9. doi: 10.1007/s10654-005-7835-x 16119429

[25] Xi B, He D, Wang QJ, Xue J, Liu M, Li J. Common polymorphisms (rs2241766 and rs1501299) in the ADIPOQ gene are not associated with hypertension susceptibility among the Chinese. Mol Biol Rep. 2012 Sep; 39(9):8771–5. doi: 10.1007/s11033-012-1739-0 22714914

[26] Fan W, Qu X, Li J, Wang X, Bai Y, Cao Q, et al. Associations between polymorphisms of the ADIPOQ gene and hypertension risk: a systematic and meta-analysis. Sci Rep. 2017 Feb 9; 7:41683. doi: 10.1038/srep41683 28181566 PMC5299502

[27] Li Y, Li X, Shi L, Yang M, Yang Y, Tao W, et al. Association of adiponectin SNP+45 and SNP+276 with type 2 diabetes in Han Chinese populations: a meta-analysis of 26 case-control studies. PLoS One. 2011 May 11; 6(5):e19686. doi: 10.1371/journal.pone.0019686 21589658 PMC3092748

[28] Tanida M, Shen J, Horii Y, Matsuda M, Kihara S, Funahashi T, et al. Effects of adiponectin on the renal sympathetic nerve activity and blood pressure in rats. Exp Biol Med (Maywood). 2007 Mar; 232(3):390–7. 17327472

[29] Wildman RP, Mancuso P, Wang C, Kim M, Scherer PE, Sowers MR. Adipocytokine and ghrelin levels in relation to cardiovascular disease risk factors in women at midlife: Longitudinal associations. Int J Obes (Lond). 2008 May; 32(5):740–8. doi: 10.1038/sj.ijo.0803782 18180784 PMC4030603

[30] Chow WS, Cheung BM, Tso AW, Xu A, Wat NM, Fong CH, et al. Hypoadiponectinemia as a predictor for the development of hypertension: a 5-year prospective study. Hypertension. 2007 Jun; 49(6):1455–61. doi: 10.1161/HYPERTENSIONAHA.107.086835 17452504

[31] Yang W, Xiao J, Yang Z, Ji L, Jia W, Weng J, et al. China National Diabetes and Metabolic Disorders Study Investigators. Serum lipids and lipoproteins in Chinese men and women. Circulation. 2012 May 8; 125(18):2212–21. doi: 10.1161/CIRCULATIONAHA.111.065904 22492668

[32] Fomenko DV, Gorokhova LG, Panev NI, Kazitskaia AS, Bondarev OI. [Clinical and experimental studies of metabolic response to chronic exposure to coal dust]. Med Tr Prom Ekol. 2011; (2):15–21. . Russian. 21506373

[33] Ma J, Wu Z, Zha X, Zhu X, Li W, Jiang M, et al. The combined effect of serum cystatin C and dyslipidemia on hypertension in a large health check-up population in China. Clin Exp Hypertens. 2019; 41(8):702–7. doi: 10.1080/10641963.2018.1545845 30497286

[34] de Lombera Romero F, Fernández Casares S, Gascueña Rubia R, Lázaro M, Hernández Simón P, Saavedra Falero J, et al. [Hypertension and dyslipidemia]. Rev Esp Cardiol. 1998; 51 Suppl 4:24–35. . Spanish. 9883066

[35] Chen J, Chen MH, Guo YL, Zhu CG, Xu RX, Dong Q, et al. Plasma big endothelin-1 level and the severity of new-onset stable coronary artery disease. J Atheroscler Thromb. 2015; 22(2):126–35. doi: 10.5551/jat.26401 25195814

[36] Zhang Y, Li S, Xu RX, Guo YL, Wu NQ, Zhu CG, et al. Distribution of High-Density Lipoprotein Subfractions and Hypertensive Status: A Cross-Sectional Study. Medicine (Baltimore). 2015 Oct; 94(43):e1912. doi: 10.1097/MD.0000000000001912 26512616 PMC4985429

[37] Paynter NP, Sesso HD, Conen D, Otvos JD, Mora S. Lipoprotein subclass abnormalities and incident hypertension in initially healthy women. Clin Chem. 2011 Aug; 57(8):1178–87. doi: 10.1373/clinchem.2011.167544 21700954 PMC3153909

[38] da Silveira CG, Di Domenico M, Hilário Nascimento Saldiva P, Ramos Rhoden C. Subchronic air pollution exposure increases highly palatable food intake, modulates caloric efficiency and induces lipoperoxidation. Inhal Toxicol. 2018 Aug–Aug; 30(9–10):370–80. doi: 10.1080/08958378.2018.1530317 30384793

[39] Pineda-Tenor D, Berenguer J, García-Broncano P, Jiménez-Sousa MA, Fernández-Rodríguez A, Diez C, et al. Association of adiponectin (ADIPOQ) rs2241766 polymorphism and dyslipidemia in HIV/HCV-coinfected patients. Eur J Clin Invest. 2014 May; 44(5):453–62. doi: 10.1111/eci.12250 24528335

[40] Zhao T, Zhao J. Genetic effects of adiponectin on blood lipids and blood pressure. Clin Endocrinol (Oxf). 2011 Feb; 74(2):214–22. doi: 10.1111/j.1365-2265.2010.03902.x 21054480

[41] de Luis DA, Izaola O, Primo D, Gómez-Hoyos E, Ortola A, López-Gómez JJ, et al. Role of rs1501299 variant in the adiponectin gene on total adiponectin levels, insulin resistance and weight loss after a Mediterranean hypocaloric diet. Diabetes Res Clin Pract. 2019 Feb; 148:262–7. doi: 10.1016/j.diabres.2017.11.007 29154912

[42] Aller R, Izaola O, Primo D, de Luis DA. The effect of single-nucleotide polymorphisms at the ADIPOQ gene locus rs1501299 on metabolic parameters after 9 mo of a high-protein/low-carbohydrate versus a standard hypocaloric diet. Nutrition. 2019 Sep; 65:44–9. doi: 10.1016/j.nut.2019.02.012 31029921

[43] Su M, Jia A, He Y, Song Y. Associations of the Polymorphisms in ADIPOQ with Circulating Levels of Adiponectin and Lipids: A Meta-Analysis. Horm Metab Res. 2021 Aug; 53(8):541–61. doi: 10.1055/a-1543-6362 34384110

[44] Zhang J, Li L, Song P, Wang C, Man Q, Meng L, et al. Randomized controlled trial of oatmeal consumption versus noodle consumption on blood lipids of urban Chinese adults with hypercholesterolemia. Nutr J. 2012 Aug 6; 11:54. doi: 10.1186/1475-2891-11-54 22866937 PMC3489577

[45] Raimondi de Souza S, Moraes de Oliveira GM, Raggio Luiz R, Rosa G. Effects of oat bran and nutrition counseling on the lipid and glucose profile and anthropometric parameters of hypercholesterolemia patients. Nutr Hosp. 2016 Feb 16; 33(1):123–30. doi: 10.20960/nh.40 27019267

[46] Guo L, Tong LT, Liu L, Zhong K, Qiu J, Zhou S. The cholesterol-lowering effects of oat varieties based on their difference in the composition of proteins and lipids. Lipids Health Dis. 2014 Dec 5; 13:182. doi: 10.1186/1476-511X-13-182 25477248 PMC4271338

[47] Drzikova B, Dongowski G, Gebhardt E. Dietary fibre-rich oat-based products affect serum lipids, microbiota, formation of short-chain fatty acids and steroids in rats. Br J Nutr. 2005 Dec; 94(6):1012–25. doi: 10.1079/bjn20051577 16351781

