# Supplementary material for: Polymorphisms of adiponectin gene and gene–lipid interaction with hypertension risk in Chinese coal miners: A matched case-control study
Source: PLoS One. 2022 Sep 12;17(9):e0268984. doi: 10.1371/journal.pone.0268984 (PMC9467355; doi:10.1371/journal.pone.0268984)
Supplement: S1 Checklist — (DOCX) [file pone.0268984.s001.docx]

STROBE Statement—checklist of items that should be included in reports of observational studies

|  | | Item No. | | Recommendation | Page  No. | | Relevant text from manuscript |
| --- | --- | --- | --- | --- | --- | --- | --- |
| **Title and abstract** | | 1 | | (*a*) Indicate the study’s design with a commonly used term in the title or the abstract | 1 | | Line 2 to 3 |
|  |  |  |  | (*b*) Provide in the abstract an informative and balanced summary of what was done and what was found | 2 | | Lines 28 to 58 |
| Introduction | | | | | | |  |
| Background/rationale | | 2 | | Explain the scientific background and rationale for the investigation being reported | 3,4 | | Lines 59 to 102 |
| Objectives | | 3 | | State specific objectives, including any prespecified hypotheses | 4 | | Lines 103 to 104 |
| Methods | | | | | | |  |
| Study design | | 4 | | Present key elements of study design early in the paper | 5 | | Lines 115 |
| Setting | | 5 | | Describe the setting, locations, and relevant dates, including periods of recruitment, exposure, follow-up, and data collection | 4,5 | | Lines 107 to 149 |
| Participants | | 6 | | (*a*) *Cohort study*—Give the eligibility criteria, and the sources and methods of selection of participants. Describe methods of follow-up  *Case-control study*—Give the eligibility criteria, and the sources and methods of case ascertainment and control selection. Give the rationale for the choice of cases and controls  *Cross-sectional study*—Give the eligibility criteria, and the sources and methods of selection of participants | 5 | | Lines 134 to 146 |
|  |  |  |  | (*b*) *Cohort study*—For matched studies, give matching criteria and number of exposed and unexposed  *Case-control study*—For matched studies, give matching criteria and the number of controls per case | 5 | | Lines 144 to 145 |
| Variables | | 7 | | Clearly define all outcomes, exposures, predictors, potential confounders, and effect modifiers. Give diagnostic criteria, if applicable | 6 | | Lines 151 to 173 |
| Data sources/ measurement | | 8* | | For each variable of interest, give sources of data and details of methods of assessment (measurement). Describe comparability of assessment methods if there is more than one group | 7,8 | | Lines 151 to 227 |
| Bias | | 9 | | Describe any efforts to address potential sources of bias | 5, 8 | | Lines 152 to 154, and 210 to 212 |
| Study size | | 10 | | Explain how the study size was arrived at | 4,5 | | Lines 118 to 130 |
| Quantitative variables | 11 | | Explain how quantitative variables were handled in the analyses. If applicable, describe which groupings were chosen and why | | 6 | Lines 165 to 171 | |
| Statistical methods | 12 | | (*a*) Describe all statistical methods, including those used to control for confounding | | 8 | Lines 205 to 227 | |
|  |  |  | (*b*) Describe any methods used to examine subgroups and interactions | | 8 | Lines 215 to 225 | |
|  |  |  | (*c*) Explain how missing data were addressed | | 6 | Lines 168 to 173 | |
|  |  |  | (*d*) *Cohort study*—If applicable, explain how loss to follow-up was addressed  *Case-control study*—If applicable, explain how matching of cases and controls was addressed  *Cross-sectional study*—If applicable, describe analytical methods taking account of sampling strategy | | 5 | Lines 144 to 146 | |
|  |  |  | (*e*) Describe any sensitivity analyses | | N/A |  | |
| Results | | | | | | | |
| Participants | 13* | | (a) Report numbers of individuals at each stage of study—eg numbers potentially eligible, examined for eligibility, confirmed eligible, included in the study, completing follow-up, and analysed | | 4, 5 | Lines 108 to 130 | |
|  |  |  | (b) Give reasons for non-participation at each stage | | 4, 5 | Lines 108 to 130 | |
|  |  |  | (c) Consider use of a flow diagram | | N/A |  | |
| Descriptive data | 14* | | (a) Give characteristics of study participants (eg demographic, clinical, social) and information on exposures and potential confounders | | 8,9 | Line 230 to 249 | |
|  |  |  | (b) Indicate number of participants with missing data for each variable of interest | | 6 | Lines 168 to 173 | |
|  |  |  | (c) *Cohort study*—Summarise follow-up time (eg, average and total amount) | | N/A |  | |
| Outcome data | 15* | | *Cohort study*—Report numbers of outcome events or summary measures over time | | N/A |  | |
|  |  |  | *Case-control study—*Report numbers in each exposure category, or summary measures of exposure | | 8,9 | Line 230 to 249 | |
|  |  |  | *Cross-sectional study—*Report numbers of outcome events or summary measures | | N/A |  | |
| Main results | 16 | | (*a*) Give unadjusted estimates and, if applicable, confounder-adjusted estimates and their precision (eg, 95% confidence interval). Make clear which confounders were adjusted for and why they were included | | 8-12 | Lines 230 to 291 | |
|  |  |  | (*b*) Report category boundaries when continuous variables were categorized | | 6 | Lines 165 to 171 | |
|  |  |  | (*c*) If relevant, consider translating estimates of relative risk into absolute risk for a meaningful time period | | N/A |  | |

Continued on next page

| Other analyses | 17 | Report other analyses done—eg analyses of subgroups and interactions, and sensitivity analyses | 12-18 | Lines 293 to 355 |
| --- | --- | --- | --- | --- |
| Discussion | | | | |
| Key results | 18 | Summarise key results with reference to study objectives | 18 | Lines 358 to 362 |
| Limitations | 19 | Discuss limitations of the study, taking into account sources of potential bias or imprecision. Discuss both direction and magnitude of any potential bias | 22 | Lines 485 to 493 |
| Interpretation | 20 | Give a cautious overall interpretation of results considering objectives, limitations, multiplicity of analyses, results from similar studies, and other relevant evidence | 18-22 | Lines 363 to 484 |
| Generalisability | 21 | Discuss the generalisability (external validity) of the study results | 22 | Lines 487 to 490 |
| Other information | |  | | |
| Funding | 22 | Give the source of funding and the role of the funders for the present study and, if applicable, for the original study on which the present article is based | N/A |  |

*Give information separately for cases and controls in case-control studies and, if applicable, for exposed and unexposed groups in cohort and cross-sectional studies.

**Note:** An Explanation and Elaboration article discusses each checklist item and gives methodological background and published examples of transparent reporting. The STROBE checklist is best used in conjunction with this article (freely available on the Web sites of PLoS Medicine at http://www.plosmedicine.org/, Annals of Internal Medicine at http://www.annals.org/, and Epidemiology at http://www.epidem.com/). Information on the STROBE Initiative is available at www.strobe-statement.org.
